# Exercise Intolerance, Benefits, and Prescription for People Living With a Fontan Circulation: The Fontan Fitness Intervention Trial (F-FIT)—Rationale and Design

**DOI:** 10.3389/fped.2021.799125

**Published:** 2022-01-06

**Authors:** Derek L. Tran, Hannah Gibson, Andrew J. Maiorana, Charlotte E. Verrall, David W. Baker, Melanie Clode, David R. Lubans, Diana Zannino, Andrew Bullock, Suzie Ferrie, Julie Briody, Peter Simm, Vishva Wijesekera, Michelle D'Almeida, Sally E. Gosbell, Glen M. Davis, Robert Weintraub, Anthony C. Keech, Rajesh Puranik, Martin Ugander, Robert Justo, Dominica Zentner, Avik Majumdar, Leeanne Grigg, Jeff S. Coombes, Yves d'Udekem, Norman R. Morris, Julian Ayer, David S. Celermajer, Rachael Cordina

**Affiliations:** ^1^Department of Cardiology, Royal Prince Alfred Hospital, Sydney, NSW, Australia; ^2^Central Clinical School, The University of Sydney School of Medicine, Sydney, NSW, Australia; ^3^Charles Perkins Centre, Heart Research Institute, Sydney, NSW, Australia; ^4^Faculty of Medicine and Health, The University of Sydney, Sydney, NSW, Australia; ^5^School of Allied Health, Curtin University, Perth, WA, Australia; ^6^Allied Health Department, Fiona Stanley Hospital, Perth, WA, Australia; ^7^The University of Sydney Westmead Clinical School, Sydney, NSW, Australia; ^8^Heart Centre for Children, The Children's Hospital at Westmead, Sydney, NSW, Australia; ^9^Heart Research Group, Murdoch Children's Research Institute, Melbourne, VIC, Australia; ^10^School of Education, Priority Research Centre for Physical Activity and Nutrition, The University of Newcastle, Newcastle, NSW, Australia; ^11^Paediatric and Adult Congenital Cardiology, Perth Children's Hospital, Perth, WA, Australia; ^12^Department of Nutrition and Dietetics, Royal Prince Alfred Hospital, Sydney, NSW, Australia; ^13^Department of Nuclear Medicine, The Children's Hospital at Westmead, Sydney, NSW, Australia; ^14^Department of Cardiology, The Prince Charles Hospital, Brisbane, QLD, Australia; ^15^Department of Cardiology, The Royal Children's Hospital, Melbourne, VIC, Australia; ^16^NHMRC Clinical Trials Centre, The University of Sydney, Sydney, NSW, Australia; ^17^Royal North Shore Hospital, The Kolling Institute, Sydney, NSW, Australia; ^18^Paediatric Cardiac Service, Queensland Children's Hospital, Brisbane, QLD, Australia; ^19^The University of Melbourne Medical School, Melbourne, VIC, Australia; ^20^Department of Cardiology, The Royal Melbourne Hospital, Melbourne, VIC, Australia; ^21^Australian National Liver Transplant Unit, AW Morrow Gastroenterology and Liver Centre, Royal Prince Alfred Hospital, Camperdown, NSW, Australia; ^22^School of Human Movement and Nutrition Sciences, Centre for Research on Exercise, Physical Activity, and Health, The University of Queensland, Brisbane, QLD, Australia; ^23^Division of Cardiac Surgery, Children's National Hospital, Washington, DC, United States; ^24^Allied Health Collaborative and Queensland Lung Transplant Service, The Prince Charles Hospital, Brisbane, QLD, Australia; ^25^School of Health Sciences and Social Work, Griffith University, Gold Coast, QLD, Australia

**Keywords:** aerobic exercise, cardiac rehabilitation, single ventricle, congenital heart disease, telehealth, exercise intolerance, hypoplastic left heart syndrome, tricuspid atresia

## Abstract

**Background:** Despite developments in surgical techniques and medical care, people with a Fontan circulation still experience long-term complications; non-invasive therapies to optimize the circulation have not been established. Exercise intolerance affects the majority of the population and is associated with worse prognosis. Historically, people living with a Fontan circulation were advised to avoid physical activity, but a small number of heterogenous, predominantly uncontrolled studies have shown that exercise training is safe—and for unique reasons, may even be of heightened importance in the setting of Fontan physiology. The mechanisms underlying improvements in aerobic exercise capacity and the effects of exercise training on circulatory and end-organ function remain incompletely understood. Furthermore, the optimal methods of exercise prescription are poorly characterized. This highlights the need for large, well-designed, multi-center, randomized, controlled trials.

**Aims and Methods:** The Fontan Fitness Intervention Trial (F-FIT)—a phase III clinical trial—aims to optimize exercise prescription and delivery in people with a Fontan circulation. In this multi-center, randomized, controlled study, eligible Fontan participants will be randomized to either a 4-month supervised aerobic and resistance exercise training program of moderate-to-vigorous intensity followed by an 8-month maintenance phase; or usual care (control group). Adolescent and adult (≥16 years) Fontan participants will be randomized to either traditional face-to-face exercise training, telehealth exercise training, or usual care in a three-arm trial with an allocation of 2:2:1 (traditional:telehealth:control). Children (<16 years) will be randomized to either a physical activity and exercise program of moderate-to-vigorous intensity or usual care in a two-arm trial with a 1:1 allocation. The primary outcome is a change in aerobic exercise capacity (peak oxygen uptake) at 4-months. Secondary outcomes include safety, and changes in cardiopulmonary exercise testing measures, peripheral venous pressure, respiratory muscle and lung function, body composition, liver stiffness, neuropsychological and neurocognitive function, physical activity levels, dietary and nutritional status, vascular function, neurohormonal activation, metabolites, cardiac function, quality of life, musculoskeletal fitness, and health care utilization. Outcome measures will be assessed at baseline, 4-months, and 12-months. This manuscript will describe the pathophysiology of exercise intolerance in the Fontan circulation and the rationale and protocol for the F-FIT.

## Background

Most babies who are born with single ventricle physiology and are palliated with the Fontan procedure will now survive into adulthood (1). The Fontan circulation is the result of a series of staged surgical procedures that redirect venous return to the pulmonary arteries, bypassing the heart. While establishing a Fontan circulation alleviates volume loading and cyanosis, it comes at the expense of elevated central venous pressure, reduced preload, and diminished (pulsatile) pulmonary artery flow. Chronically, these abnormal hemodynamic conditions cause long-term complications, including premature mortality, Fontan-associated liver disease, protein-losing enteropathy, thromboembolic events, arrhythmias, and heart failure (2). However, major advances in surgical techniques and medical care have dramatically improved prognosis, and the projected population of people living with a Fontan circulation in Australia and New Zealand is expected to double over the next 20 years (3). This improved prognosis highlights the need to establish adequate health care services and therapies to provide appropriate care for people living with a Fontan circulation.

Exercise training is a well-established therapy and is part of routine clinical care in people with cardiopulmonary conditions (4). In non-congenital cardiac conditions, improvements in aerobic exercise capacity—reflected by peak oxygen uptake (VO_2_)—following cardiac rehabilitation or exercise training is associated with better prognosis and clinical outcomes, including potential reductions in mortality and hospitalization (5–9). While this association has yet to be directly shown in people with a Fontan circulation, it would seem plausible that increasing peak VO_2_ with exercise training would yield similar benefits in this cohort, especially since the peripheral muscle pump is of heightened importance in this unique physiological environment. This is supported by studies that show an association between higher aerobic exercise capacity and better prognosis in people with a Fontan circulation (10–13).

Multiple series from tertiary centers around the world have shown the utility of peak VO_2_ to identify high-risk phenotypes. People who have congenital heart disease (CHD) with a peak VO_2_ below 15.5 ml/kg/min are at a 2.9-fold increased risk of hospitalization or death compared to those with greater aerobic exercise capacity (10). Higher peak VO_2_ is also associated with better end-organ function in people with a Fontan circulation (14). Furthermore, deterioration in aerobic exercise capacity appears to be the strongest predictor of adverse events in people with a Fontan circulation (15, 16).

Given the apparent prognostic implications associated with higher aerobic exercise capacity, it would seem intuitive to understand the pathophysiology of exercise intolerance and optimize therapies such as exercise training that can improve peak VO_2_. This manuscript will review the pathophysiology of exercise intolerance in the Fontan circulation and describe the rationale, aims, and methods for the multi-center, randomized, controlled Fontan Fitness Intervention Trial (F-FIT).

## Pathophysiology of Exercise Intolerance in the Fontan Circulation

### Cardiopulmonary Exercise Testing Response in the Fontan Circulation

**Figure 1:**
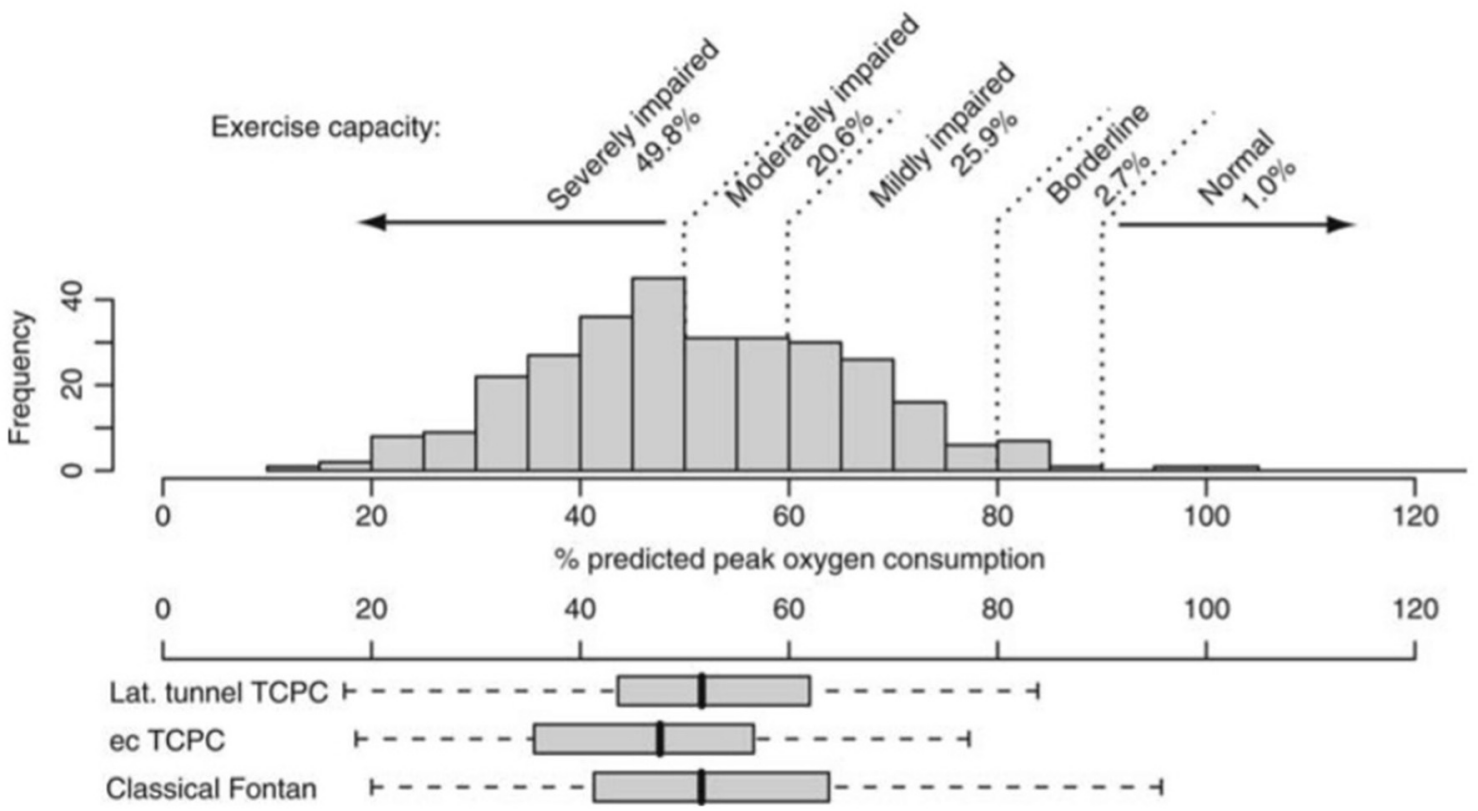
Distribution of % predicted peak oxygen uptake (consumption) in patients after Fontan operation and its distribution in patients with different types of Fontan surgery. ec, extracardiac; lat, lateral; TCPC, total cavopulmonary connection. Reproduced from (12).

People living with a Fontan circulation usually have at least moderately impaired aerobic exercise capacity (Figure 1), with large series reporting an average peak VO_2_ ranging from 23 to 27 ml/kg/min (52-61% predicted) (12, 13). The typical cardiopulmonary exercise testing response includes a depressed peak heart rate (HR), elevated minute ventilation (V_E_)/carbon dioxide production (VCO_2_) slope (ventilatory inefficiency), reduced peak work rate, and increased breathing frequency (17–19). Peak oxygen pulse—a surrogate for stroke volume and arteriovenous oxygen extraction—is impaired, with an early plateau or downsloping trajectory of the oxygen pulse curve, likely reflecting cardiogenic (preload) limitation to exercise performance and intrinsic skeletal muscle dysfunction (20, 21). Exercise ventilatory oscillation is also common (22). Furthermore, a subset of people may also have mechanical ventilatory limitations reflected by limited breathing reserve (23). Interestingly, the anaerobic threshold and other submaximal measures of exercise capacity are often better preserved compared to peak VO_2_, albeit still lower than normal predicted values. Some people may also experience exercise-induced oxygen desaturation secondary to diffusion type limitation or right-to-left shunting *via* veno-venous collaterals or Fontan fenestration.

### Serial Changes in Aerobic Exercise Capacity

The change in aerobic exercise capacity over time may be more prognostically significant than a single measurement (16); a decline in peak VO_2_ is associated with increased risk of adverse cardiovascular events, death, and cardiac transplantation (15, 24). Some longitudinal studies have reported an average decline in aerobic exercise capacity ranging from 0.8 to 2.6 percentage points per year (15, 24–26). The accelerated rate of decline may result in a premature deterioration below the “critical” peak VO_2_ threshold (16.6 ml/kg/min or 50% predicted) that significantly increases the risk of adverse events (27, 28). This may explain the high prevalence of morbidity, and premature mortality observed as early as the third or fourth decade of life. Promisingly, more recent reports have shown slower rates of decline or even an increase in aerobic exercise capacity trajectory (29). Understanding the contributors to the trajectory of aerobic exercise capacity may aid the determination of appropriate therapies and potentially identify the appropriate timing for intervention.

### Surgical Factors

#### Age at Fontan Completion

The optimal age at Fontan completion remains controversial. It is uncertain whether prolonging the period prior to partial surgical correction (Glenn shunt) can allow for optimal pulmonary vascular development, albeit at the expense of cyanosis and volume loading. Fontan completion at a later age may also allow for a larger conduit to “optimize” flow. However, data appears to support the notion of early Fontan completion to preserve long-term aerobic exercise capacity (30, 31). This association may be explained by protecting the single ventricle from excessive volume loading with earlier age at Fontan completion.

#### Type of Circulation

**Figure 2:**
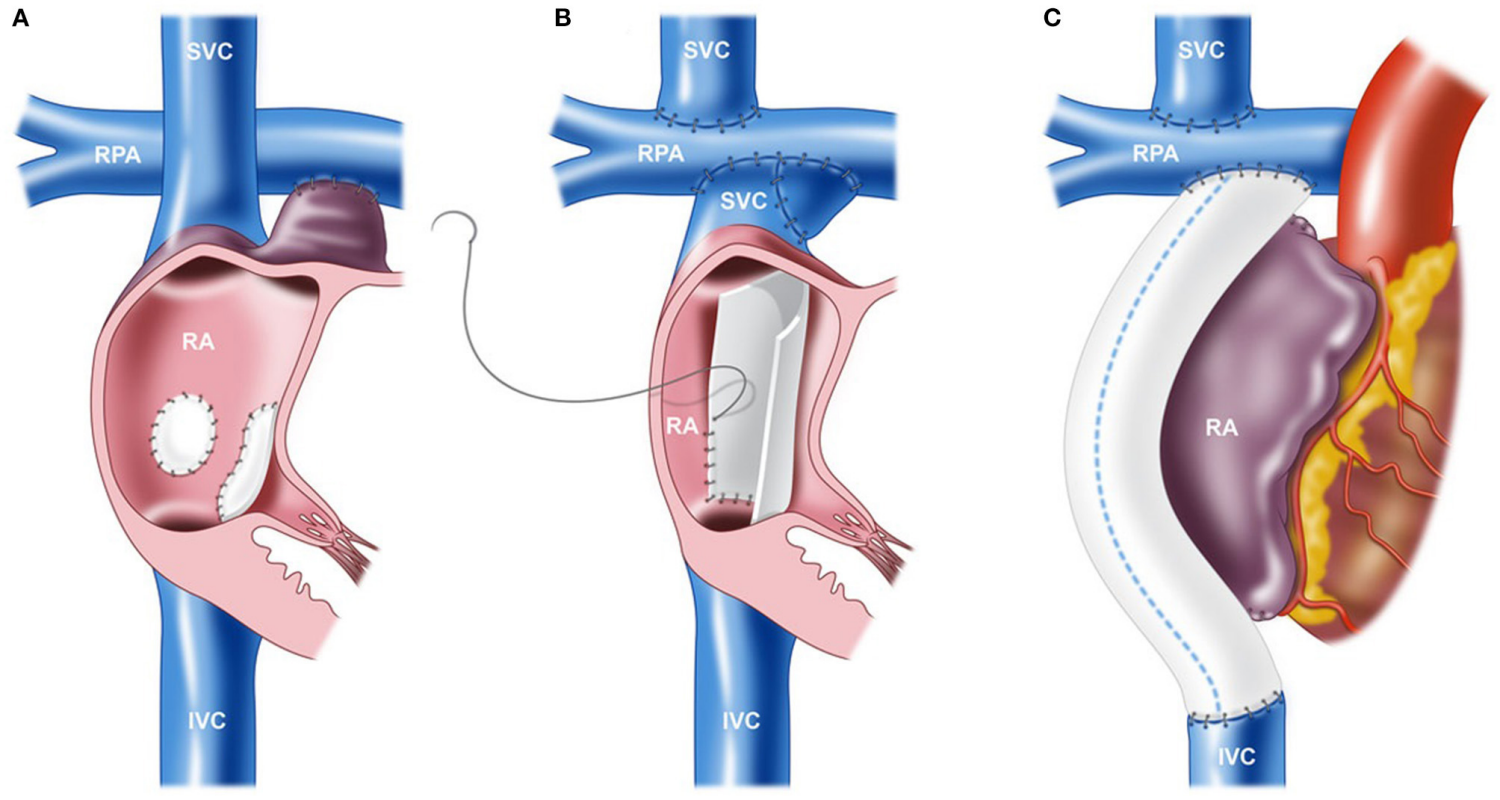
Various techniques of the Fontan procedure. **(A)** Atriopulmonary connection. **(B)** Lateral tunnel total cavopulmonary connection (TCPC). **(C)** Extracardiac conduit TCPC. IVC, inferior vena cava; RA, right atrium; RPA, right pulmonary artery; and SVC, superior vena cava. Reproduced from (32).

Since the original atriopulmonary connection-type Fontan procedure, there have been various modifications to this approach (Figure 2). While the notion of the original procedure was to “ventriculize” the right atrium to compensate as a subpulmonary pump, long-term follow-up data demonstrated poor prognostic outcomes with this surgical approach (33). The preferred approach in the current era is the total cavopulmonary connection (lateral tunnel or extracardiac conduit), which has dramatically improved long-term outcomes and survival because atriopulmonary connections are more prone to arrhythmias, cardiac maladaptation (heart failure), worse atrial and ventricular mechanics, and premature mortality (1, 33, 34). Importantly, the modification to the total cavopulmonary connection-type Fontan circulation has optimized hemodynamics and flow energetics. Indeed, those with a total cavopulmonary connection tend to have greater pulmonary flow and stroke volume compared to those with an atriopulmonary connection, but the impact on aerobic exercise capacity is unclear. In adolescents and adults, peak VO_2_ and submaximal exercise measures were higher in those with a total cavopulmonary connection (35), although surprisingly, large seminal series show no difference in aerobic exercise capacity between groups (12, 26). These contradictory findings may be attributed to the vastly heterogeneous group of patients that present with extensive variations in arrhythmia burden, ventricular morphology, pulmonary vascular function, muscle mass, lung function, physical activity levels, and ventricular function. It is likely that the type of Fontan circulation is associated with aerobic exercise capacity only in a selected subset of older patients with other co-existing complications and suboptimal Fontan circuit geometry.

### Cardiac Factors and Pulmonary Vascular Resistance: Implications for Cardiac Output

#### Cardiac Factors

##### Systolic Ventricular Function

At rest, cardiac output is often within the normal range or only mildly depressed in the single ventricle circulation, with relatively well-preserved systolic function (contractility). Reductions in resting cardiac output may be the consequence of “ventricular-vascular” uncoupling instead of impaired contractility (36). However, at elevated HRs, there is evidence of limited inotropic response, likely secondary to decreased preload rather than intrinsic cardiac abnormalities (37). Although some studies have reported a correlation between measures of systolic function and aerobic exercise capacity (38, 39), the degree of ventricular systolic dysfunction does not completely account for the level of exercise intolerance experienced in the majority of the cohort, most of whom have preserved systolic function. This is supported by our local data (35) and a large, multi-center series from the Pediatric Heart Network, which did not find any association between systolic ventricular function and peak VO_2_ (40). Furthermore, if contractility was a significant cause of exercise intolerance, the use of inotropic agents would theoretically improve cardiac output, and in turn, aerobic exercise capacity, which has not been demonstrated in the Fontan circulation (41). In summary, while it is probable that systolic ventricular function contributes to aerobic exercise capacity, it is unlikely to be a primary contributor to exercise intolerance unless systolic ventricular dysfunction is severe (42).

##### Diastolic Ventricular Function

In the Fontan circulation, preserving diastolic function is imperative to minimize pulmonary pressure and increase pulmonary blood flow. Even modest elevations in filling pressure can have significant effects on preload and aerobic exercise capacity (28). Resting diastolic dysfunction assessed by echocardiogram inversely correlates with peak VO_2_ (38, 43, 44). Of note, however, studies that utilize traditional echocardiography measures of ventricular function (systolic and diastolic) should be interpreted with caution, as they are poorly validated and likely inaccurate in the setting of preload deprivation and atypical chamber geometry in the Fontan circulation (45, 46). While the ability of the ventricle to “pull” blood through the pulmonary vasculature is limited, preserving diastolic function in the single ventricle is likely an important factor to prevent the deterioration of aerobic exercise capacity by maintaining ventricular filling.

##### Dominant Ventricle Morphology

The myocardial architecture and coronary blood supply of the left ventricle are designed to sustain the systemic circulation (47). Unsurprisingly, the ability of the right ventricle to support the systemic circulation is often suboptimal, and adverse remodeling likely ensues over time in many patients, which theoretically should impair aerobic exercise capacity. Indeed, some studies have reported an association between left ventricular morphology and aerobic exercise capacity (25, 48).

However, series involving older cohorts were unable to detect an association between exercise intolerance and dominant ventricle morphology (26, 49–51). This is consistent with other clinical measures of exercise capacity (6-min walk distance or treadmill exercise duration), which showed no differences in exercise performance between ventricular morphology type in patients with an extracardiac conduit type circulation (52). The latest Pediatric Heart Network study also did not show an association between ventricular morphology and peak aerobic exercise capacity, although patients with a dominant left ventricle had better submaximal exercise capacity (higher VO_2_ at anaerobic threshold) (26). Notably, even those with a dominant left ventricle show evidence of pathological abnormalities compared to the normal biventricular heart (53), and may in part, explain the conflicting findings reported.

Despite the association between ventricular morphology and aerobic exercise capacity previously reported, people with a systemic right ventricle can still achieve normal or even supranormal exercise capacity (14, 54), suggesting that it is not a central limiting factor. This is likely related to the somewhat limited role that contractility has on cardiac output in the setting of limited preload.

##### Outflow Obstruction and Valvular Regurgitation

Outflow obstruction may be attributed to valvular stenosis, aortic obstruction, subvalvular stenosis (e.g., membrane or muscle bar), or supravalvular stenosis, which may impede the augmentations of cardiac output during exercise. This can lead to increased afterload, ventricular hypertrophy, and potentially maldistribution of blood flow, with significant implications on atrial filling pressures and stroke volume. Importantly, these obstructions may progress over time and can become dynamic with exertion (55), further restricting flow and potentially resulting in a precipitous decline in cardiac output during exercise.

Regurgitation of the systemic semilunar or atrioventricular valve predisposes the heart to chamber enlargement and progressive ventricular dysfunction, which may impair aerobic exercise capacity. Ohuchi *et al*. reported that a small subset of Fontan patients with atrioventricular valve insufficiency had lower peak VO_2_ (48). This reduction in aerobic exercise capacity may be associated with the deleterious consequences that accompany atrioventricular valve regurgitation (e.g., elevations in atrial pressure), which affect the transpulmonary flow gradient. However, more prospective data are required to confirm the degree to which atrioventricular valve insufficiency contributes to reduced aerobic exercise capacity.

##### Chronotropic Response

**Figure 3:**
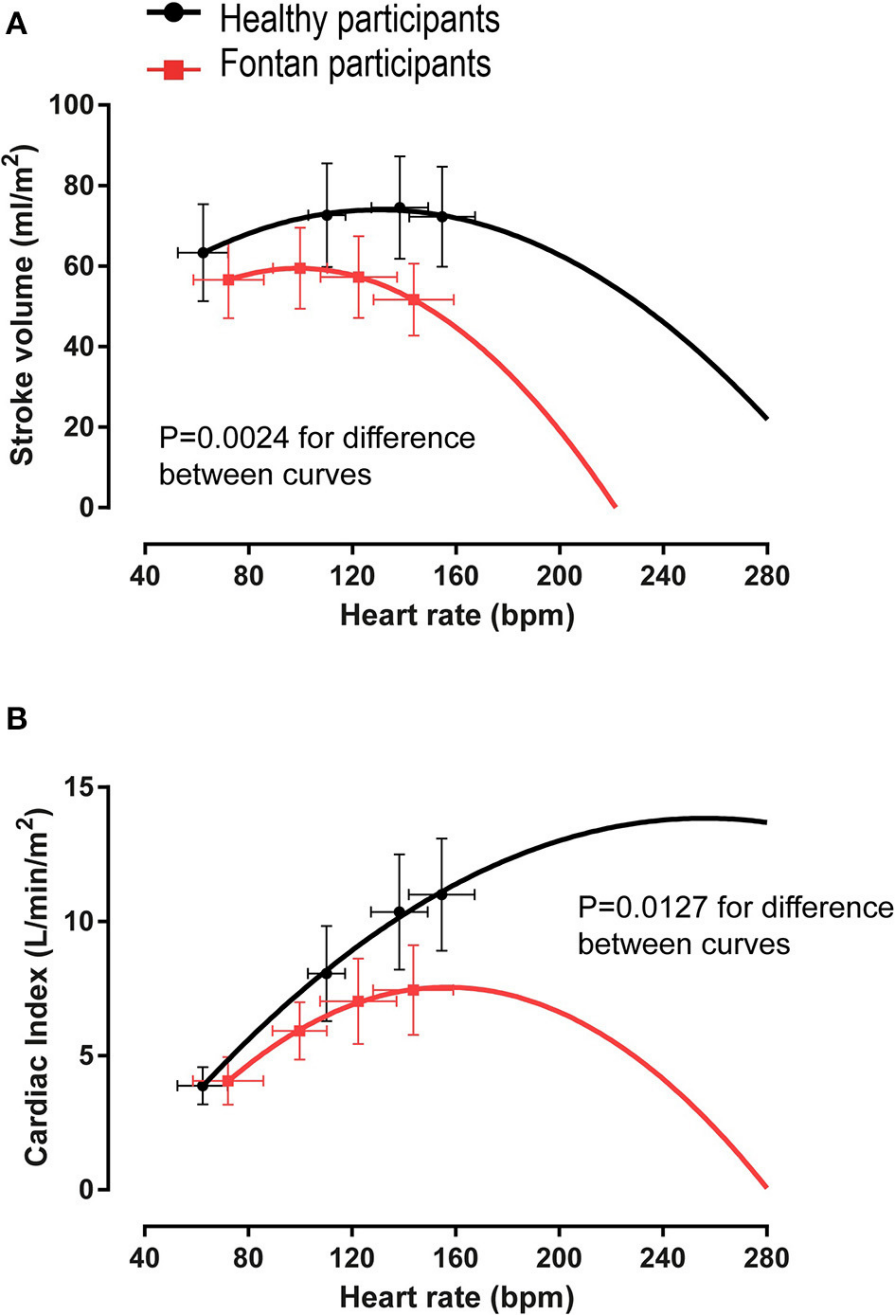
Quadratic regression analysis of mean stroke volume and cardiac output vs. average heart rate values. In people with a Fontan circulation, an additional increase in heart rate beyond peak exercise values would result in **(A)** a disproportionate fall in stroke volume such that **(B)** cardiac output cannot increase further. Modified from (56).

Chronotropic limitation is a common factor associated with impaired aerobic exercise capacity in people with cardiac conditions. In contrast to patients with a biventricular circulation, mildly-to-moderately depressed peak HR (often described as “chronotropic incompetence”) may be, in part, an autoregulatory response to reduced preload. Scarring of the conduction system related to cardiac surgery, intrinsic developmental abnormalities of the conduction system, and drugs (such as beta-blockers and anti-arrhythmic medications) also impair the chronotropic response. Unless chronotropic limitation is severe, peak HR does not appear to significantly impair aerobic exercise capacity. Further supporting this notion, there is no difference in chronotropic limitation between patients who achieve “normal” aerobic exercise capacity compared to those with reduced aerobic exercise capacity (54). In addition, atrial pacing studies have shown no improvement in cardiac output. Further increases in peak exercise HR may result in a plateau or decrease in cardiac output and promote exercise intolerance (Figure 3). The observed diminished HR reserve (HRR) can be predominantly attributed to hemodynamic abnormalities (i.e., reduced preload) (56). However, during relative submaximal exercise intensities, the chronotropic response is appropriate or even higher compared to healthy control subjects (57).

#### Pulmonary Vasculature

##### The “Critical Bottleneck”

Gewillig et al. have usefully described the pulmonary vascular bed as the “critical bottleneck” that is predominantly responsible for impeding ventricular filling and cardiac output, which in turn impairs aerobic exercise capacity (58). Transpulmonary flow restriction attributed to inadequate pulmonary artery growth and progressive pulmonary vascular disease likely ensues from the absence of pulsatile pulmonary flow; pulmonary artery growth essentially ceases after Fontan completion, potentially restricting venous return and impairing aerobic exercise capacity. Supporting this notion, pulmonary artery size is inversely correlated with New York Heart Association Functional Class and is positively associated with peak VO_2_ (59). Maldistribution of pulmonary blood flow, which is common in Fontan physiology due to altered branch pulmonary artery anatomy and flow dynamics, is also associated with decreased aerobic exercise capacity (60).

**Figure 4:**
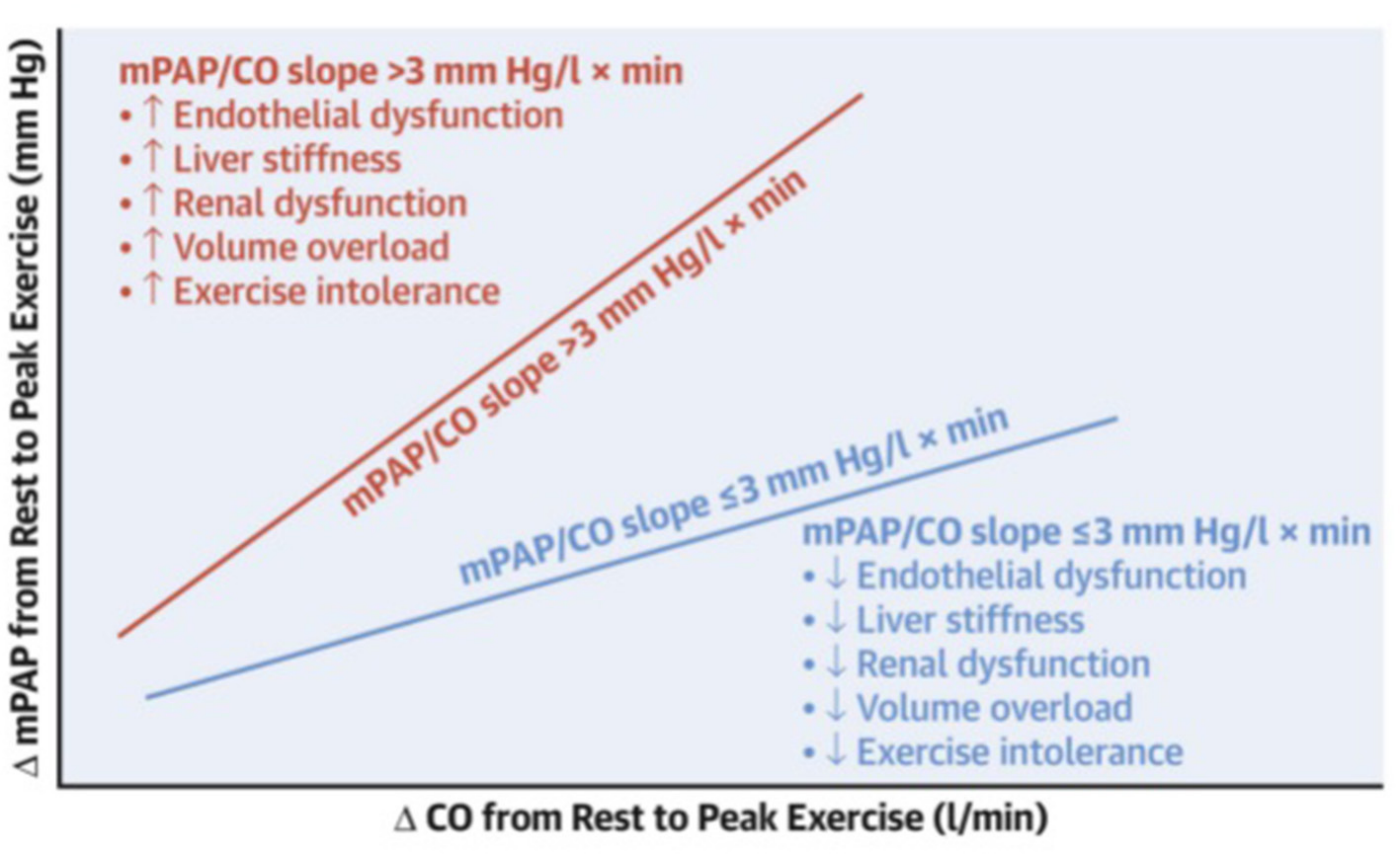
Schematic showing the relationship between pulmonary vascular reserve (VR) and end-organ function. Pressure-flow relationship showing change in mean pulmonary artery pressure (mPAP) (Fontan pressure) per unit change in cardiac output (CO), or mPAP/CO slope, during exercise. Abnormal pulmonary VR defined as mPAP/CO slope > 3 (red) is associated with worse endothelial dysfunction and end-organ dysfunction (more liver stiffness, renal dysfunction, volume overload, and exercise intolerance) as compared to normal pulmonary VR defined as mPAP/CO slope ≤ 3 (blue). Reproduced from (61).

The influence of the pulmonary vasculature on aerobic exercise capacity has been elegantly demonstrated in an invasive study performed by the Mayo Clinic (Figure 4) (61). Egbe et al. showed that people with abnormal exercise pulmonary vascular reserve (primarily reflecting pulmonary vascular dysfunction) have significantly worse aerobic exercise capacity (49% predicted peak VO_2_) compared to those with a normal pulmonary vascular reserve (67% predicted peak VO_2_). When interpreted with other hemodynamic data (decreased stroke volume index with increased pulmonary vascular resistance index), it is reasonable to speculate that the difference in peak VO_2_ is attributed to lower pulmonary vascular resistance, resulting in enhanced ventricular filling in patients with normal pulmonary vascular reserve.

It is likely that opening the “critical bottleneck” (i.e., reducing pulmonary vascular resistance) would theoretically improve ventricular filling and aerobic exercise capacity, but therapies targeted at the pulmonary vasculature have yielded disappointing and inconsistent results; a 7% increase was the greatest improvement in peak VO_2_ reported (62–64). Marginal improvements were also observed for submaximal exercise parameters (VO_2_ at anaerobic threshold) with phosphodiesterase five inhibitors; the landmark FUEL (Fontan Udenafil Exercise Longitudinal) trial failed to show improvements in peak VO_2_ (62). Disappointingly, the reported treatment effect (3-5%) with pulmonary vasodilator therapies for peak VO_2_ is of questionable clinical benefit (65).

Despite statistically insignificant improvements in peak aerobic exercise capacity, drug therapies such as phosphodiesterase five inhibitors may still provide clinical benefits (66). Long-term use of pulmonary vasodilators may reduce systemic venous pressure and attenuate or prevent future complications or decline in aerobic exercise capacity.

The available data suggest that the pulmonary vascular characteristics are an important contributor to aerobic exercise capacity in the Fontan circulation. However, treatments that target pulmonary vasculature alone are insufficient to “normalize” aerobic exercise capacity—perhaps because the bottleneck is fixed and/or unlike pulmonary arterial hypertensive vasculopathy—or perhaps simply because trials are underpowered and more careful patient selection is required due to the vast heterogeneity of the cohort.

### Extending Beyond the Heart and Pulmonary Vasculature

#### Lung Function

Typically, resting lung function demonstrates a mildly restrictive ventilatory pattern in people who have a Fontan circulation (67–69). The impairments in lung function parameters are associated with lesion complexity, multiple sternotomies or thoracotomies, physical activity restriction, respiratory muscle dysfunction, body mass index (BMI), and scoliosis (19, 67, 70). Reduced forced vital capacity, lung volumes, and diffusion capacity of the lung for carbon monoxide are common and associated with decreased peak VO_2_ (67, 70–72). In addition to better aerobic exercise capacity, superior lung function is associated with increased handgrip strength that may reflect superior respiratory muscle strength (73).

Interestingly, despite the apparent resting lung function abnormalities, in general, patients rarely encroach upon their breathing reserve (74). However, in an extensive series of 260 young people with a Fontan circuit, 23% of those with impaired aerobic exercise capacity (<80% predicted peak VO_2_) had limited breathing reserve, suggesting a mechanical ventilatory limitation to exercise (23). Assessing ventilatory limitation using breathing reserve alone likely underappreciates the pulmonary contribution to exercise intolerance. The addition of tidal flow-volume loops during exercise testing may reveal further underlying ventilatory constraints. Indeed, studies that utilized inspiratory capacity maneuvers to assess dynamic operating lung volumes and expiratory flow limitations during exercise show further evidence of abnormal ventilatory responses (75). At submaximal work rates, people with Fontan physiology have lower inspiratory reserve volumes compared to controls, possibly resulting in higher elastic work of breathing. These ventilatory abnormalities during exercise may be attributed to the restrictive ventilatory impairment observed and likely contributes to the heightened dyspnea intensity reported at submaximal workloads (75).

The relationship between lung function and aerobic exercise capacity may also be explained by additional mechanisms. Reductions in forced vital capacity may be of particular importance, as it can impair the ability to compensate for ventilatory inefficiency during exercise (23). Furthermore, it has been postulated that smaller lungs have less blood volume and reduced capacitance, and in turn, diminished capacity to accommodate decreases in pulmonary vascular resistance, which has important implications for ventricular filling during exercise. While it is clear that lung function contributes to exercise intolerance in the single ventricle circulation, the extent and precise mechanisms remain poorly defined.

#### Systemic Vascular Resistance and Vascular Function

Vascular dysfunction (increased arterial stiffness and endothelial dysfunction) is associated with worse aerobic exercise capacity (76–78). This is likely related to impaired skeletal oxygenation and muscle blood flow rather than the contribution of endothelial dysfunction to increased systemic vascular resistance (elevated afterload) in the setting of limited preload reserve (41, 79). Elevated systemic vascular resistance is likely a secondary phenomenon required to maintain adequate blood pressure in the Fontan circulation at rest and during exercise (80).

#### Sex

Data from the Australian and New Zealand Fontan Registry has shown that the male sex is associated with an increased risk of premature death or transplantation (1, 81). Consistent with this observation in people with a Fontan circulation, male sex is a factor associated with lower aerobic exercise capacity (relative to age and sex) and progressive exercise intolerance (26, 49). The sex differences in aerobic exercise capacity may be related to reduced muscle mass compared to healthy controls, which is likely more pronounced in males (especially during puberty), and the inability of the single ventricle to support the increased metabolic demands of the greater absolute skeletal muscle mass in males.

#### Hypoxemia and Cyanosis

Cyanosis or hypoxemia is common in people with a Fontan circulation. It is unclear whether a patent fenestration is associated with improved aerobic exercise capacity as a result of increased ventricular filling or if the establishment of a right-to-left shunt and subsequent hypoxemia will impair it; data on the effects of fenestration closure are inconsistent (82–84). However, fenestration closure has been shown to improve ventilatory efficiency (83), which may decrease dyspnea perception during submaximal exercise.

Paradoxically, lower hemoglobin is associated with better aerobic exercise capacity (14). A similar relationship was reported with lean mass, which is inversely correlated with hemoglobin (85). This is probably because elevated hemoglobin reflects a compensatory erythrocytosis in the setting of low oxygen saturation. This contrasts with the findings of Kodama et al., who reported a positive correlation between peak VO_2_ and hemoglobin (86). Regardless of the reported conflicting associations, the contribution of arterial desaturation to reduced aerobic exercise capacity is minimal, explaining <5% of the variance in peak VO_2_ (40).

#### Skeletal Muscle Function

Handgrip strength, dynamic muscular strength, and muscular endurance have all been reported to be lower in people with a Fontan circulation than in healthy, age-matched controls and are associated with reduced skeletal muscle mass (85, 87–89). In a series with a heterogenous sample of CHD lesions (30% Fontan), when strength was indexed to lean mass, there was no difference in isometric strength compared with healthy controls (90). This may suggest that the reductions in muscle strength reported can largely be attributed to the reduction in lean mass that we and others have demonstrated (85, 89, 91). Beyond generalized muscle weakness (92), peripheral skeletal muscle blood flow and ergoreceptor function appear to be abnormal (89, 93), and this is likely accompanied by a shift in muscle fiber type (to type IIb), similar to the findings in acquired heart failure. Furthermore, impaired skeletal muscle oxidative capacity has been shown using MRS P^31^ spectroscopy, and delayed muscle oxygen uptake kinetics denote potential muscle metabolic abnormalities (21, 94). The combination of these skeletal muscle abnormalities likely result in the early onset of metabolic acidosis, premature fatigue during exercise, and consequently impaired aerobic exercise capacity.

#### Body Composition

##### Skeletal Muscle Mass

Even in relatively young people with a Fontan circulation, there is a high prevalence of skeletal muscle deficit compared to age-sex matched controls (21, 91). Although myopenia (low muscle mass) is prevalent across many CHD lesions, it is likely those with a Fontan circulation experience a greater degree of lean mass deficits. The causes of lean muscle deficits are poorly defined, but in the Fontan circulation, relative deconditioning, chronically elevated central venous pressure, physical inactivity, neurohormonal activation, and altered blood flow are likely contributing factors. To highlight this pathophysiological difference to sarcopenia (age-related muscle deficits) and low lean mass in other CHD lesions, Tran et al. described the term Fontan-associated myopenia (appendicular lean mass index Z-score < −2) (85). Low lean mass in the setting of Fontan physiology is particularly concerning, given the strong correlation between skeletal muscle mass and exercise stroke volume and/or aerobic exercise capacity (21, 89, 95, 96). This can be attributed, inter alia, to improved cardiac preload—greater skeletal muscle mass decreases venous compliance and squeezes a greater volume of blood back toward the pulmonary vasculature and heart (97). Leg muscle contractions may also generate a pulsatile flow profile in the pulmonary vascular bed (98).

##### Obesity and Adiposity

A higher BMI is associated with lower aerobic exercise capacity in people with a Fontan circulation. High levels of adiposity—particularly in the thoracic region—may impair the function of the respiratory bellows. The Pediatric Heart Network Fontan III study showed that patients in the lowest tertile, based on percent predicted peak VO_2_, were more likely to be overweight or obese (26). However, defining “healthy” weight status using BMI in this cohort is problematic because the high prevalence of lean mass deficiency conceals the presence of increased fat mass when BMI is used as a surrogate of adiposity (85). Although the adverse effects of obesity will likely impair aerobic exercise capacity (14), further research using reference measures of lean and fat mass should be conducted to better characterize the implications of obesity on Fontan physiology.

#### Respiratory Muscle Dysfunction

Extending beyond the effects that respiratory muscle weakness may have on dynamic lung function, it also contributes to an increase in motor command output, resulting in a greater sensation of breathlessness during exercise (99). Furthermore, in the setting of limited cardiac reserve, the redirection of blood flow from the exercising skeletal muscles to the respiratory muscles (“metaboreflex”) promotes premature fatigue and profound impairment in aerobic exercise capacity. These mechanisms may, in part, explain the association between respiratory muscle function and aerobic exercise capacity in Fontan patients (92).

At rest, Fontan physiology is heavily dependent on respiration to promote ventricular filling. Theoretically, it would be expected that improving inspiratory muscle strength would augment the respiratory muscle pump and ventricular filling. While inspiratory muscle training has been shown to improve ventilatory efficiency and resting cardiac output, most studies have not resulted in statistically significant increases in peak VO_2_ (100–102). This may be because the skeletal muscle pump accounts for the majority of the increase in cardiac output, with only minor contributions attributed to the respiratory pump during exercise (103). Respiratory muscle training may be beneficial in patients who specifically have clinical respiratory muscle weakness. This notion was supported by a recent randomized controlled trial, where baseline measures of maximal inspiratory pressure indicated inspiratory muscle weakness in the cohort; peak VO_2_ increased after 4 months of inspiratory muscle training (104).

#### Benefits of Exercise Training and Safety

Paradoxically, the most effective non-invasive therapy to manage exercise intolerance is exercise training (97, 105, 106). A recent review of respiratory muscle and exercise training studies in over 200 people with a Fontan circulation showed that the majority of studies resulted in improvements in peak VO_2_ (107), and increases of up to 23% (treatment effect 30%) have been shown with combined aerobic exercise and light resistance training (104). Other benefits included improvements in skeletal muscle mass, cardiac output, peripheral muscle oxygenation, and ergoreceptor function (97, 105, 106, 108–110). Importantly, some studies also show improvements in health-related quality of life (111–115).

Furthermore, long-term participation in sports, physical activity, or exercise may have direct benefits on Fontan physiology. Increasing skeletal muscle mass through resistance exercise training can enhance the function of the peripheral muscle pump and augment venous return (108). The periodic increase in volume load during exercise “stretches” the preload deprived ventricle and may attenuate the phenomenon of progressive “disuse hypofunction” (58, 116). Regular physical activity like exercise training transiently but repetitively increases pulsatile flow and recruit pulmonary vessels, which may have important implications for pulmonary vascular growth and function (97, 116). Indeed, those who participate in regular physical activity (particularly during childhood) appear to have better Fontan physiology and are more likely to exhibit a high physical performance (“Super-Fontan”) phenotype (54, 117, 118). Collectively, these mechanisms may explain the association between higher peak VO_2_ and better end-organ function, clinical outcomes, and prognosis (10, 13, 14). However, further research is required to confirm this hypothesis.

##### Safety of Physical Activity and Exercise Training

Reviews of exercise training studies in people with a Fontan circulation have not reported any serious adverse events associated with exercise training (107, 110). Undeniably, during exercise, systemic venous pressure can increase dramatically in the Fontan circulation (119). While there are some concerns related to the deleterious effects of the transient elevation of central venous pressure during exercise on end-organ function, the current evidence suggests these are unwarranted. Higher aerobic exercise capacity is associated with healthier end-organ function biomarkers, potentially reflecting decreased venous pressure, better hemodynamics, and reduced hepatic congestion (14, 54). This is further supported by a series that showed lower venous pressure and better markers of end-organ function in adult patients who increased their aerobic exercise capacity during childhood (reflecting increased physical activity levels or exercise training) (118). Together, these data should alleviate the concerns regarding safety and end-organ damage associated with chronic moderate-to-vigorous intensity exercise training, but more prospective data are needed.

## Addressing the Unanswered Questions: Rationale for the Fontan Fitness Intervention Trial—Therapies and Future Direction

**Figure 5:**
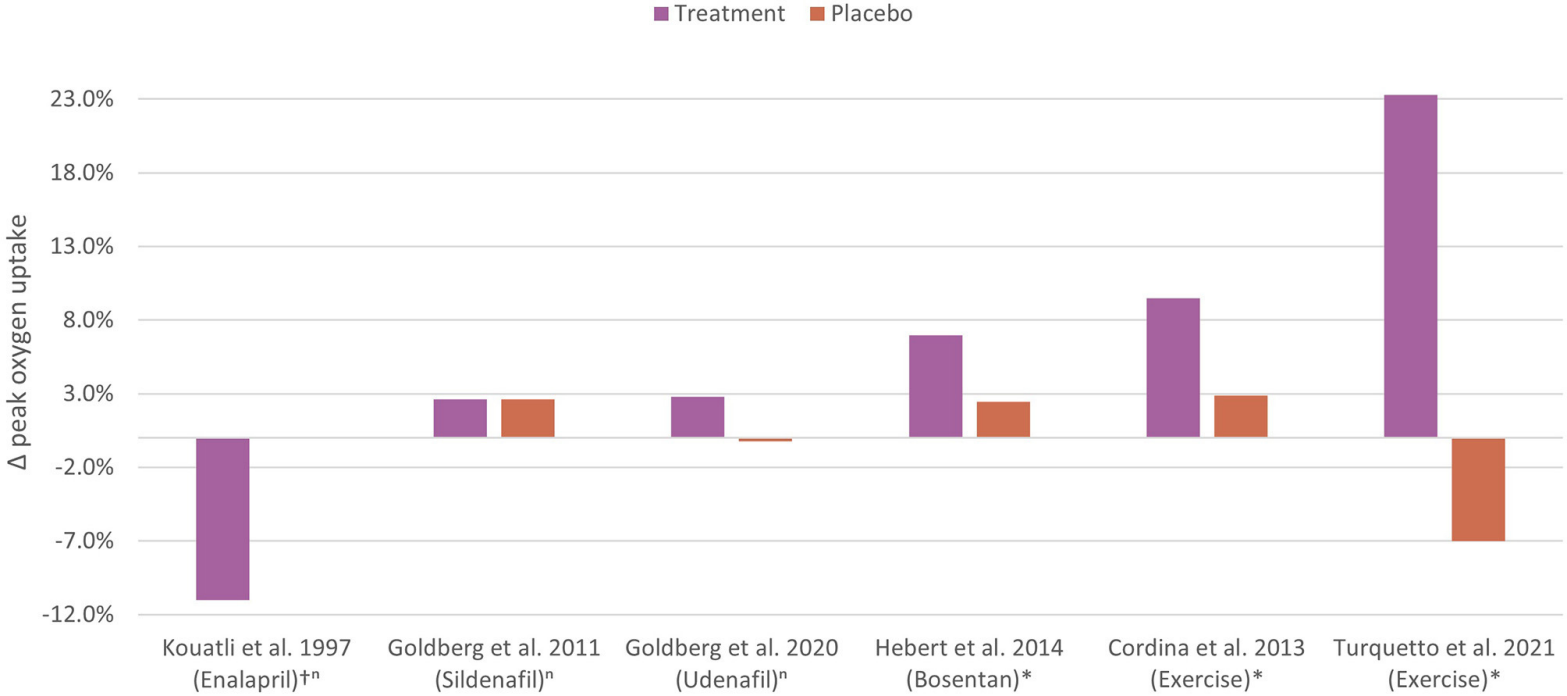
Controlled trials of non-invasive therapy to improve aerobic exercise capacity in the Fontan circulation. The percentage change in peak oxygen uptake following non-invasive therapies or placebo in Fontan cohorts are shown. The most effective non-invasive therapy is exercise training. n, non-significant; *, statistically significant; ^†^, percentage difference between groups. Kouatli et al. (120); Goldberg et al. (63); Goldberg et al. (62); Hebert et al. (64); Cordina et al. [high intensity resistance training] (108); Turquetto et al. [combined aerobic exercise and light resistance training] (104).

The mechanisms underlying impaired aerobic exercise capacity in the setting of Fontan physiology differ significantly from other chronic cardiac conditions. Traditional pharmacotherapies used to manage exercise intolerance in the biventricular circulation are of limited utility in the Fontan circulation. Currently, exercise training has been shown to be the most effective, non-invasive therapy for improving aerobic exercise capacity in people who have a Fontan circulation (Figure 5) (105, 106, 109).

**Figure 6:**
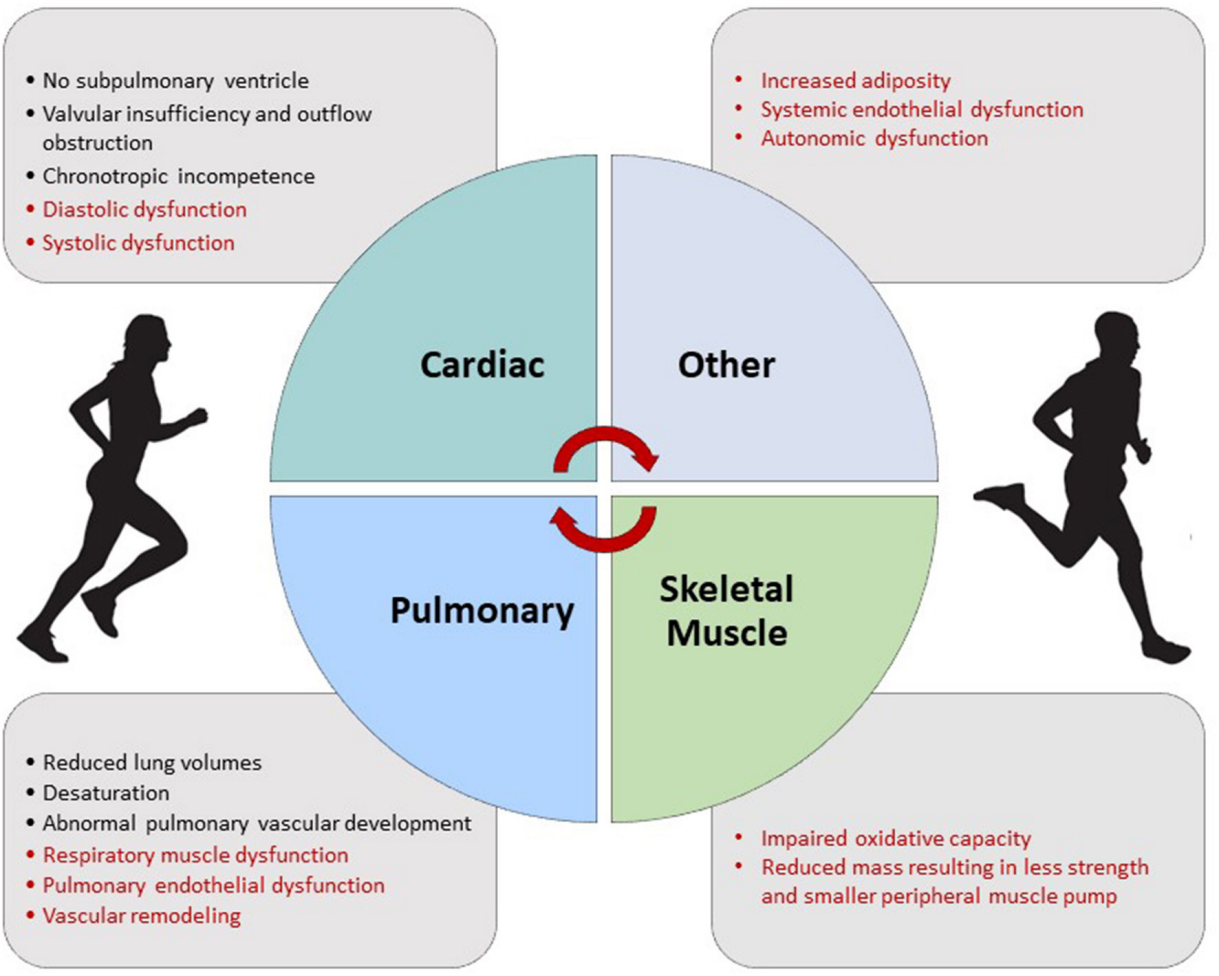
Factors contributing to exercise intolerance in the Fontan circulation. Factors in red may be improved with exercise training.

While this review identifies common factors that contribute to exercise intolerance (Figure 6), understanding factors associated with superior aerobic exercise capacity is also important to more deeply characterize the pathophysiology. Recent series have studied cohorts of people with Fontan physiology with normal or superior aerobic exercise capacity (14, 54, 121). We previously described a subset of young people who can achieve normal or even supranormal exercise performance (“Super-Fontan”) (121). Importantly, in this series, a proportion had unfavorable Fontan features (e.g., dominant right ventricle, pacemakers, and atriopulmonary connections), suggesting extracardiac factors play a significant role in aerobic exercise capacity.

A common characteristic of a person with a Fontan circulation who has “normal” aerobic exercise capacity is regular participation in moderate-to-vigorous intensity physical activity from a young age (54, 121, 122). Some possible mechanisms for this observation may include superior development of the pulmonary vasculature (e.g., increased pulmonary artery size), increased lower limb lean mass, higher lung volumes due to stronger respiratory muscles, and adaptive remodeling of the single ventricle due to better preload (97, 98, 106). Exercise training can systemically target components that are both distal (downstream) and proximal (upstream) to the critical “bottleneck” as well as the pulmonary vasculature itself. This makes exercise training an attractive therapy and may explain the efficacy observed compared to drug therapies that may only target a single component of Fontan physiology.

However, despite the aforementioned benefits associated with exercise training and physical activity, traditionally, most people with CHD have not received formal advice (beyond restrictions) on physical activity, sports, and exercise training during their clinical consultations (123). This may be related to the paucity of quality evidence available on safety and efficacy; most studies are based on small heterogenous samples and were without a control group. While current exercise training recommendations are available to guide clinical practice (106, 109, 124, 125), these are predominantly based on clinical experience and expert opinion. Indeed, the most recent 2018 AHA/ACC and 2020 ESC guidelines suggest that there is only moderate (level B) evidence to support recommending cardiac rehabilitation or exercise training in people with CHD (4, 126). Therefore, adequately powered, multi-center, randomized, controlled trials such as the F-FIT are required to provide high-quality (level A) evidence to conclusively support recommending exercise training in clinical practice.

Furthermore, the “traditional” model of exercise training for people living with chronic diseases requires face-to-face supervision by exercise professionals (at least initially) that are often only available at expert centers. Whilst it is likely that performing exercise training in a supervised fitness facility setting is “optimal”, this method of exercise training delivery requires high resource utilization and may not be economically feasible or practical for many people living with a Fontan circulation. In addition, traditional exercise training programs offered to people with CHD are usually designed for older adults with chronic conditions (e.g., cardiac rehabilitation), which may not be suitable for the relatively young adult CHD population. Indeed, previous studies have identified this as a potential barrier to participation (127, 128), and over half of those surveyed with CHD expressed interest in a technology-directed, home-based, exercise program (129).

The F-FIT will be one of the first phase III multi-center, randomized, controlled trials to provide high-quality evidence to “optimize” exercise training in people living with a Fontan circulation. The F-FIT will also investigate if a telehealth exercise training model (that requires less resources) can produce equivalent (non-inferior) results to a traditional supervised gym-facility-based approach.

The primary objectives of the F-FIT are to:

a) Establish the efficacy of a 4-month traditional supervised gym-based aerobic and resistance exercise training program of moderate-to-vigorous intensity on peak VO_2_ compared to usual care in adolescents and adults.
b) Establish the efficacy of a 4-month physical activity program of moderate-to-vigorous intensity on peak VO_2_ compared to usual care in children.
c) Evaluate if a 4-month telehealth exercise training program of moderate-to-vigorous intensity can produce comparable (non-inferior) improvements in peak VO_2_ compared to the traditional exercise training group in adolescents and adults.

Secondary objectives include:

a) Determining if participants in the exercise intervention groups can maintain changes in peak VO_2_ with remote support over an 8-month period.
b) To evaluate the health economics (cost-effectiveness) of exercise training interventions based on health-related quality of life, health care utilization, and patient costs.
c) To characterize the mechanisms that underlie changes in peak VO_2_.
d) To characterize the physiological and neurocognitive changes associated with exercise training, including changes in cardiopulmonary testing measures, peripheral venous pressure, body composition (skeletal muscle mass, fat mass, and bone mineral density), endothelial function, neurohormonal activation, skeletal muscle oxygenation, respiratory muscle and lung function, neurocognitive and neuropsychological function, metabolites, nutritional and dietary status, liver stiffness, and cardiac function.

## Study Design and Methods

### Study Population

Participants will be recruited from the Australian and New Zealand Fontan Registry (130), National CHD Database, and eight quaternary CHD centers, including Royal Prince Alfred Hospital, Sydney, Australia; The Children's Hospital at Westmead, Sydney, Australia; Royal Melbourne Hospital, Melbourne Australia; Royal Children's Hospital, Melbourne Australia; Perth Children's Hospital, Perth, Australia; Fiona Stanley Hospital, Perth, Australia; The Prince Charles Hospital, Brisbane, Australia; and Queensland Children's Hospital, Brisbane, Australia. Advertisements will also be disseminated *via* social media, websites, and flyers to facilitate recruitment.

The F-FIT will include people with a Fontan circulation aged 10-55 years. Participants will also need to be ≥6 months post-Fontan completion, clinically stable, and on stable medical therapy for ≥3 months to be eligible. The inclusion and exclusion criteria are provided in Table 1. A two-step exclusion process will take place:

1) Based on the patient's most recent medical records, phone screening, and approval from their treating cardiologist.
2) Following baseline testing.

**Table 1:** Study inclusion and exclusion criteria.

**Inclusion criteria**
•People with a Fontan circulation aged 10-55 years •Medically stable and on stable medical therapy for ≥3 months •≥6 months post-Fontan completion
**Exclusion criteria**
•Planned intervention within 2 years •Mental or physical disability that prevents participation in exercise training •Current or actively planned pregnancy •Uncontrolled systemic hypertension at rest or during exercise •Clinically unstable or recent significant change in therapy (within 3 months) •Physiological stage D in accordance with ACC/AHA guidelines •COVID-19 unvaccinated individuals despite being eligible according to ATAGI recommendations •Unreliable internet connection •Current participation in structured sports or exercise training for more than 30 min, three times a week

Participants will be excluded prior to randomization during the two-step eligibility evaluation if any of the following are identified: categorized as physiological stage D, have a planned surgical intervention within 2 years, current pregnancy or actively planned pregnancy (within 1 year), mental or physical disability that restricts participation in exercise training, uncontrolled arrhythmias or (systemic) hypertension at rest or during exercise, a recent significant change in medical therapy (<3 months), and people who currently participate in structured sports or exercise training for more than 30 min, three times per week. People who are COVID-19 unvaccinated and are eligible for vaccination according to the Australian Technical Advisory Group on Immunization (ATAGI) recommendations will also be excluded.

### Randomization and Stratification

The F-FIT will involve three arms in adolescents and adults (≥16 years), and two arms in children (<16 years). Adolescents and adults will be randomized in an allocation of 2:2:1 to either a traditional gym-based exercise program (traditional group), a telehealth exercise training program (telehealth group), or usual care (control group), respectively. Children will be randomized to either an exercise training program or usual care (control group) based on a 1:1 allocation. Randomization will be stratified by: baseline aerobic exercise capacity (<65% or ≥65% predicted peak VO_2_); sex (male or female); and age (16-34 years or 35-55 years for adolescents and adults; 10-12 years or 13-15 years for children). Computer-generated, random permuted blocks will be prepared by an independent statistician in the Clinical Epidemiology and Biostatistics Unit (CEBU) at Murdoch Children's Research Institute (MCRI) and incorporated into the REDCap randomization tool (hosted by MCRI) that will be embedded in the REDCap database created for this trial. The randomization schedules will be prepared for each recruitment site.

### Study Investigations

**Figure 7:**
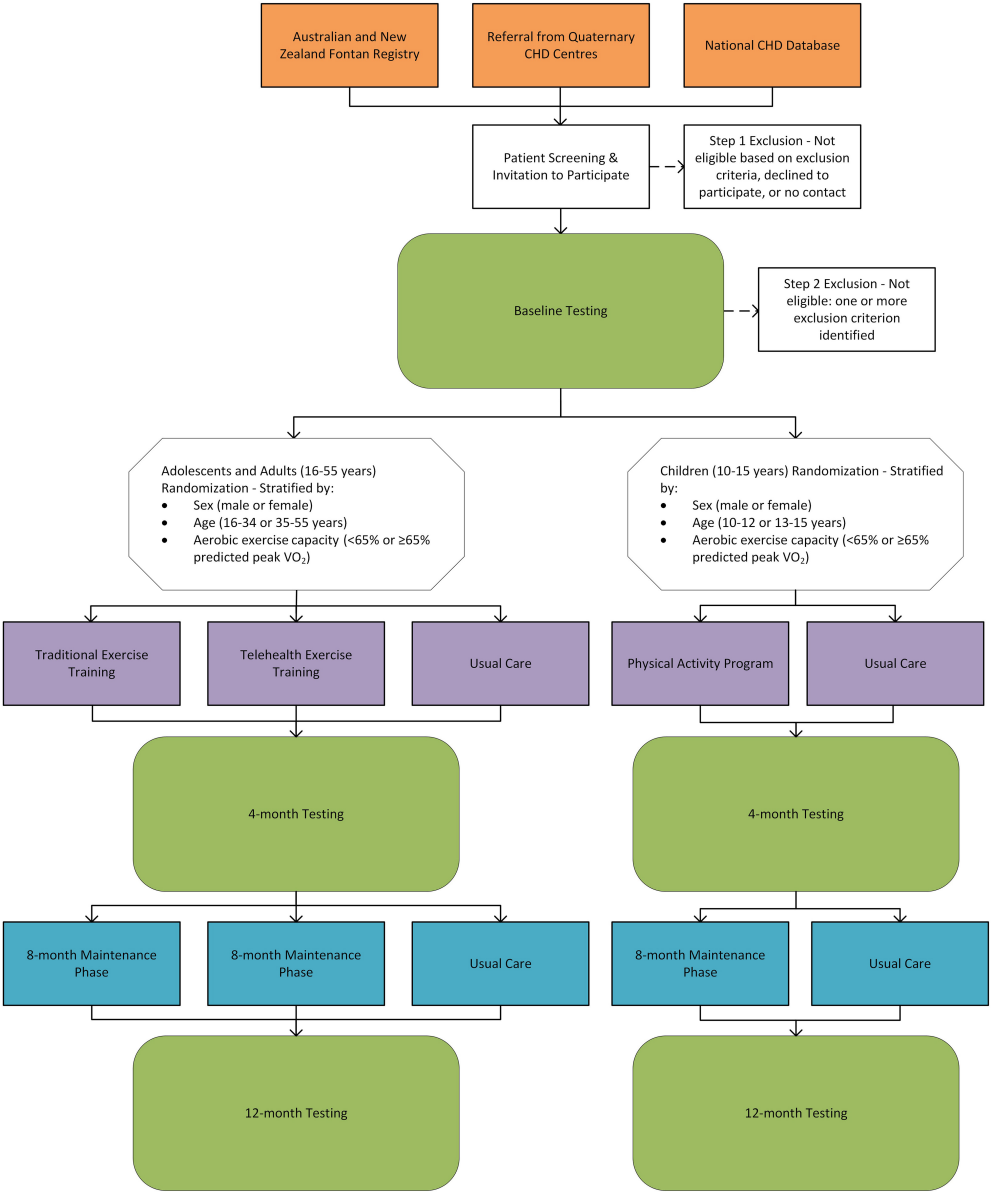
The Fontan Fitness Intervention Trial (F-FIT) study design flow diagram.

The F-FIT will conduct a range of assessments at baseline, 4-months, and 12-months. The study design is outlined in Figure 7, and the assessments are summarized in Table 2. In brief, all participants will undergo a detailed evaluation of aerobic exercise capacity, respiratory muscle and lung function, body composition, musculoskeletal fitness, endothelial function, quality of life, neurocognitive and neuropsychological function, neurohormonal activation, liver stiffness, dietary intake and nutritional status, metabolites, habitual physical activity levels, and cardiac function. Follow-up visits will be conducted within 15 days of the scheduled reassessment date. If a testing date cannot be scheduled within 15 days, participants in the intervention groups may continue exercise training in accordance with the protocol to prevent detraining for up to 31 days.

**Table 2:** Assessments and testing.

	**Outcomes measures**	
Cardiopulmonary exercise testing	Aerobic exercise capacity (peak VO_2_)^‡^, V_E_/VCO_2_ ratio and slope, RER, HR, OUES, VO_2_ at AT, work rate, oxygen pulse, VO_2_/work rate slope, and peripheral venous pressure	
Respiratory muscle and lung function tests	FEV_1_, FVC, FEV_1_/FVC ratio, TLC, DL_CO_, PImax, and PEmax	
Dual-energy x-ray absorptiometry	Lean mass, fat mass, bone mineral content, and bone mineral density^†^	
Liver elastography	Liver stiffness	
Near-infrared spectroscopy^†^	HHb, HbO_2_, and skeletal muscle oxidative capacity	
Neurocognitive function assessment (Cogstate)	Psychomotor function, attention, visual learning and memory, verbal learning and memory, processing speed, social-emotional cognition, working memory, and executive function scores	
Habitual physical activity (accelerometers; Actigraph GT9X Link)	Counts per minute, steps per day; and time spent in sedentary, light, moderate, vigorous, and moderate-to-vigorous activity	
Nutrition and dietary assessments (ASA24, SGA^†^ or SGNA^†^, GSRS^†^, and indirect calorimetry^†^)	SGA (in adults)/SGNA (in children) classification of nutritional status; GSRS (reflux, abdominal pain, indigestion, diarrhea, constipation scores, and total score); dietary macronutrient and micronutrient intake and composition, and REE	
Flow-mediated dilation (FMD)^†^	FMD% (Δ diameter), baseline diameter, peak diameter, and time to peak	
Laboratory and biochemical investigations	NT-proBNP and metabolomic analysis	
Transthoracic echocardiography	AVV S/D ratio, valvular function, VTI, annulus size, aortic flow, and ventricular function	
Resting and exercise cardiac MRI^†^	Ventricular volumes (end-diastolic, end-systolic, stroke volume), ejection fraction, flows (aortic, vena caval), diastolic function (feature tracking, T1 mapping E'), pulmonary artery size (Nakata index), lung water density, hepatic T1 mapping, and AV valve function	
Anthropometry and BIA	Height, weight, waist circumference, BMR, total body water, %BF, and skeletal muscle index	
Quality of life (PedsQL core and cardiac modules)	Physical functioning, emotional functioning, social functioning, school/work functioning, psychosocial functioning, heart problems and treatment, perceived physical appearance, treatment anxiety, cognitive problems, communication and total scores	
	**Adolescents and adults**	**Children**
Musculoskeletal fitness testing	Chest press 1RM, leg press 1RM, number of leg press repetitions at 70% 1RM (muscular endurance), and handgrip strength	Number of sit-ups, number of push-ups, standing long jump distance, and handgrip strength
Health economic analysis (EQ-5D-5L, CHU-9D, patient cost, and health care expenditure data linkage)	Health state in EQ-5D dimensions, patient cost, and health care utilization	CHU-9D scores, patient cost, and health care utilization

‡ *Primary outcome*,

† *Conducted in a subset of participants at selected sites. 1RM, one-repetition maximum; AT, anaerobic threshold; AV, atrioventricular; ASA24, automated self-administered dietary assessment tool; AVV S/D, atrioventricular systolic to diastolic duration; BIA, bioelectrical impedance analysis; BMR, basal metabolic rate; BF, body fat; DL_CO_, diffusing capacity of the lung for carbon monoxide; FEV_1_, forced expiratory volume in one second; FMD, flow-mediated dilation; FVC, forced vital capacity; GSRC, gastrointestinal symptom rating scale; HbO_2_, oxyhemoglobin; HHb, deoxyhemoglobin; HR, heart rate; MRI, magnetic resonance imaging; NT-proBNP, N-terminal pro b-type natriuretic peptide; OUES, oxygen uptake efficiency slope; PEmax, maximum static expiratory pressure; PImax, maximum static inspiratory pressure; REE, resting energy expenditure; RER, respiratory exchange ratio; SGA, subjective global assessment; SGNA, subjective global nutritional assessment; TLC, total lung capacity; VTI, velocity time integral; VE/VCO2, ventilatory equivalent for CO2; VO_2_, oxygen uptake*.

### Statistical Considerations

#### Power Analysis

Sample size calculations have accounted for a 10% dropout over 4-months.

##### Adolescents and Adults

This study will involve testing two hypotheses: the first involves demonstrating the superiority of the traditional exercise training group compared to the usual care group with regards to improvements in peak VO_2_. The second involves demonstrating that telehealth exercise training is non-inferior to traditional exercise training. Using this rationale, we based the sample size calculation for a three-arm trial with a randomization allocation of 2:2:1 using the R package Three *Armed Trials* (version 1.0-3).

We will require 110 adolescent and adult Fontan participants (44 each in the traditional and telehealth training groups and 22 in the usual care group) to achieve at least 80% power (two-sided α of 5%) to detect a difference of 10% in peak VO_2_ (standard deviation of 5%) between the traditional exercise training group compared to the control group. If there is a statistically significant difference between the traditional and control group (i.e., *p*-value < 0.05), then the test of non-inferiority will have 80% power (one-sided α of 2.5%) of showing that telehealth training retains at least 85% of the effect seen in traditional testing compared to control.

##### Children

A total of 70 children with a Fontan circulation (35 in the training group and 35 in the usual care group) is required to achieve >99% power (two-sided α of 5%) to detect a difference in peak VO_2_ of 10 ± 5% at 4-months.

#### Statistical Analysis

The primary analysis will be based on intention-to-treat (ITT), including all randomized participants regardless of exposure to the allocated treatment or adherence to the trial protocol. Comparison of the primary outcome measure (change in peak VO_2_ at 4-months) between the groups will be estimated using linear regression adjusted for the stratification factors used during randomization. Results will be presented as the difference of means with a corresponding 95% confidence interval (CI) and *p*-value. Secondary outcomes at 4-months and 12-months will be compared between the groups using linear regression with adjustment for the stratification factors for continuous outcomes and binary regression adjusted for the stratification factors used during randomization for binary outcomes where results will be presented as a risk difference and corresponding 95% CI.

For each participant cohort, if the proportion of missing data for the primary outcome is more than 5%, analysis based on multiple imputation may be performed. A sensitivity analysis to compare the results of analyses restricted to participants with complete data and analyses where those with missing data are included using multiple imputation will be performed. If used, multiple imputation models will be conducted for the outcome variable, and 50 completed data sets will be imputed by chained equations, including all the participants initially randomized. The primary outcome, randomization strata variables and variables predictive of (i) missingness and/or (ii) the change in peak VO_2_ will be included in the imputation model.

### Exercise Training and Physical Activity Interventions

#### Exercise Training Interventions for Adolescents and Adults

##### Traditional Fitness Facility-Based Exercise Training

Patients randomized to the traditional model of exercise training delivery will participate in moderate-to-vigorous intensity aerobic and resistance exercise training three times a week for 4-months. All sessions will be supervised by a qualified exercise professional (e.g., exercise physiologists or and physiotherapists) in small groups of 1-4 people. The sessions will be supervised in a local fitness facility near the participant's residence, where they will be provided with a complimentary membership for the duration of the study. Participants will start and conclude each session with a 5 min warm-up and cool down, which may include low-intensity exercise and dynamic or static stretching. The structure of the sessions will involve 10 min of aerobic exercise, 30 min of resistance exercises, followed by another 10 min of aerobic exercise.

The first 10-min bout of aerobic exercise will be performed on a cycle ergometer, and the second 10-min bout is selected based on the participant's preference to allow for autonomy. Aerobic exercise training will start at a moderate intensity (40-50% HRR) and progress up to vigorous intensity (70-80% HRR) after 10 weeks, as tolerated. The aerobic exercise training work rate will be continually adjusted throughout the program to maintain the target training HR range.

Resistance training will comprise of 5 exercises, including the leg press, seated row, leg curl, chest press, and calf raise. The participant's one-repetition maximum (1RM) will be assessed for each exercise during the first session and every 4 weeks to titrate the load to the appropriate training intensity. Resistance exercise intensity will start at 3 sets of 8-12 repetitions at 60% 1RM and be progressed to 70% 1RM after 2 weeks. Participants will be provided with ~60 s rest between sets. Consistent with clinical practice, intensity can be up titrated based on the participant's rating of perceived exertion (RPE) and observer RPE for aerobic exercise training using the OMNI scale. For resistance exercises, the two for two method, and the participant's or observer's RPE using the OMNI scale can be used to facilitate progression in between 1RM tests, which can be guided by the supervising exercise professional. An outline of the method of progression is shown in Table 3. The total estimated duration of each session is 60-75 min.

**Table 3:** Exercise training progression for the traditional group.

**Weeks (sessions)**	**Intensity category**	**Intensity**
**Aerobic exercise training progression**		
1-2 (6)	Moderate	40-50% HRR
3-6 (12)	Moderate	50-60% HRR
7-10 (12)	Vigorous	60-70% HRR
11-16 (18)	Vigorous	70-80% HRR
**Resistance exercise training progression**		
1-2 (6)	Moderate (moderate load)	60% 1RM (3 sets, 8-12 repetitions)
3-16 (42)	Vigorous (moderate-to-high load)	70% 1RM (3 sets, 8-12 repetitions)

*HRR, Heart rate reserve; 1RM, one-repetition maximum*.

##### Telehealth Exercise Training

People in the telehealth group will participate in partially supervised moderate-to-vigorous intensity aerobic and resistance exercise training 3 times a week for 4-months. Prior to each session, a 5-min warm-up and cool-down will be performed and may include low-intensity exercise and dynamic or static stretching. Participants will be provided with a Gymstick™ and HR monitor for exercise training. Participants will be asked to perform 20 min of aerobic exercise training independently three times a week, starting at 40-50% HRR and progressing to 70-80% HRR. The aerobic exercise training progression will be consistent with the traditional group shown in Table 3. Exercise training HR will be transmitted to a mobile app, and participants will be asked to record the average and maximal HR as well as their RPE and session duration of each aerobic session in a training log.

Resistance exercise sessions will be supervised by qualified exercise professionals and delivered via Zoom in groups. Participants will perform 3 sets of 8-12 repetitions of various resistance exercises using a Gymstick™ with a target RPE of 7 using the OMNI scale by week 3 for each exercise. Resistance exercises may include squats, upright rows, lunges, seated rows, chest press, and calf raises. When the participant rates an exercise lower than 7 on the OMNI scale in consecutive sets or sessions, the resistance (load) of the Gymstick™ will be increased or participants will be asked to increase their repetitions to the upper limit of the prescribed range. Similar to the traditional group, the intensity can be adjusted based on the two-for-two method and observer RPE. Participants will also be asked to record exercise session details in a training log. The total estimated time to complete both the aerobic and resistance exercise session is ~60-75 min.

#### Children's Physical Activity Program

Children allocated to the intervention group will participate in a face-to-face physical activity program once a week for 4-months. The SAAFE principles will be utilized to guide the delivery of the program in an engaging and enjoyable manner (131). Weekly sessions will be supervised by an exercise professional and conducted at a community sport center or fitness facility near the participant's residence in small groups of 3-10 participants/family members when possible. The duration of each session will be ~90 min and consist of an exercise training circuit, foundational movement skills practice, and physically active games. Prior to each session, participants will engage in a 5-10 min warm-up that includes a variety of games, aerobic exercises, and dynamic stretching.

Participants will be provided with HR monitors that will transmit their HR in real-time to an app on an electronic device (e.g., iPad, tablet, or laptop) to monitor exercise intensity during the exercise circuit. The exercise circuit will be conducted in an interval format and encompasses a combination of aerobic and resistance exercises. Exercises may include but are not limited to squats, hopping, broad jumps, push-ups, backward running, and walking lunges. The target session average HR will initially be at moderate intensity (≥40% HRR) and progress to vigorous intensity (≥70% HRR) after 10 weeks, consistent with the adolescent and adult aerobic exercise programs (Table 3). The exercise circuit will last ~30 min.

Following the exercise circuit, participants will practice a variety of foundational movement skills (e.g., kicking, catching, throwing, and hitting) for 5-10 min, which is facilitated by the exercise professional, family members, and caregivers. After the practice period, participants will engage in physically active games for ~20 min. Each session will conclude with a 5–10 min cool-down that may consist of low-intensity aerobic activities and stretching.

In addition to the weekly face-to-face physical activity sessions, participants will also be provided with a variety of tasks to complete in their own time. These tasks will be directed at promoting a healthy lifestyle or complement the physical activity sessions.

#### Exercise Training Considerations

**Figure 8:**
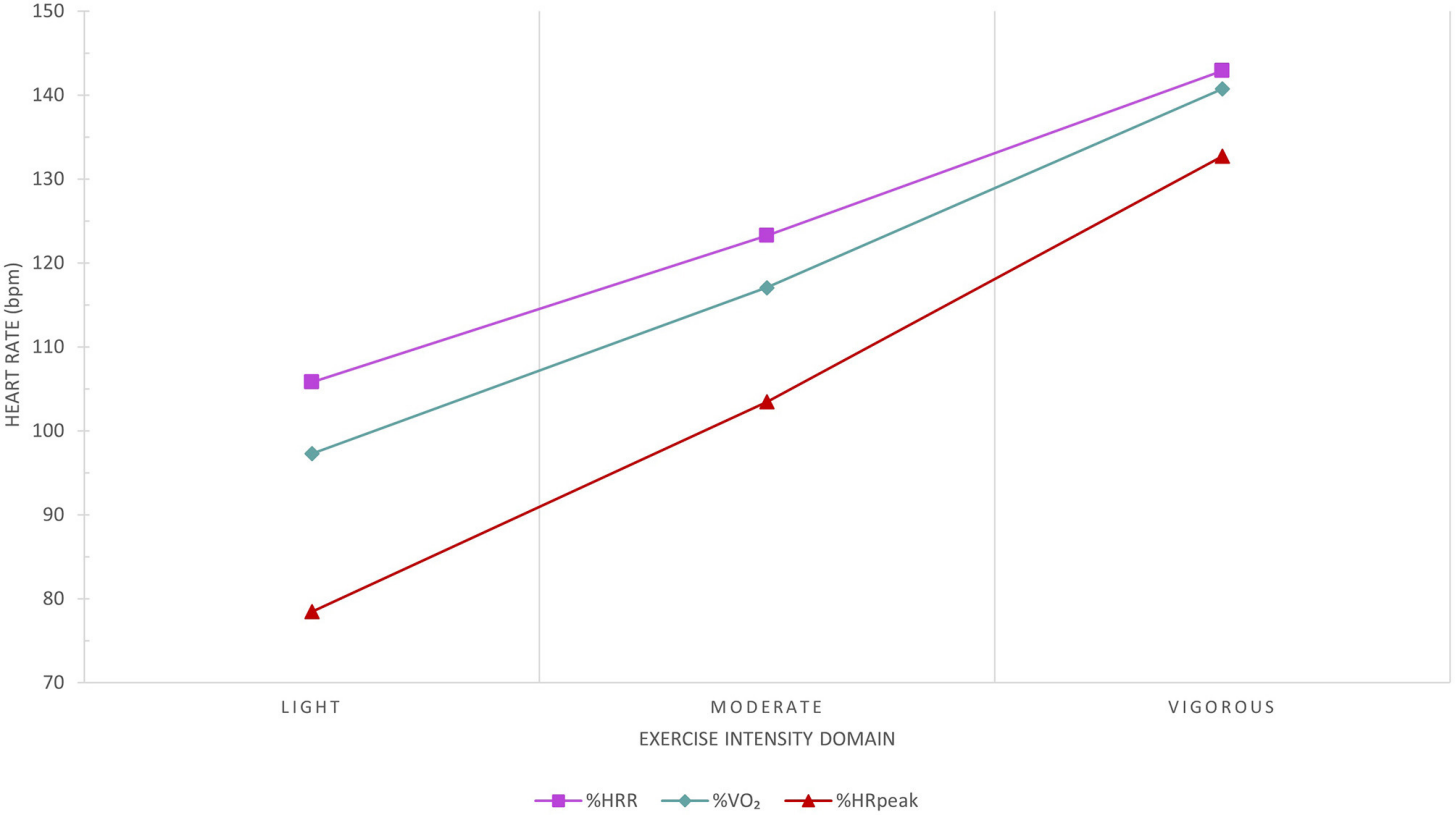
Comparison between the percentage of peak heart rate (HR; [%HRpeak]) and percentage of HR reserve (%HRR) methods to the average reference HR recorded at the corresponding percentage of peak VO_2_ (%VO_2_) exercise intensity domain. The %HRpeak method significantly underestimates the reference exercise HR. The %HRR method results in clinically insignificant differences to the corresponding reference HR recorded in all exercise intensity domains based on %VO_2_ and reflects metabolic load more accurately than the %HRpeak method. Data from 287 congenital heart disease patients at Royal Prince Alfred Hospital.

The target aerobic exercise training HR range (intensity) will be prescribed using the percentage of HRR method, which more accurately reflects metabolic load compared to prescribing aerobic exercise intensity based on the percentage of peak HR (Figure 8). The resting and peak HRs obtained at baseline cardiopulmonary exercise testing will be used for determining the prescribed target HR ranges:


$$\begin{array}{l} Exercise\;Training\;HR\;\left(HRR\;method\right)=\%\;target\;intensity\\ \times \left(peak\;HR\;-resting\;HR\right)+resting\;HR \end{array}$$

In participants who are prescribed β-adrenergic blocker agents or other HR limiting drugs, exercise testing and training should occur between 3 and 10 h after the dose was taken (132). If participants are unable to exercise at the prescribed HR range (e.g., chronotropic incompetence) or unable to tolerate aerobic exercise training in the prescribed HR range, aerobic exercise training intensity will be guided by both the observer and participant reported RPE, and the talk test. Furthermore, participants will exercise at least 10-15 beats below the ischemic or discharge threshold in people with stable ischemia or for those who have an implantable cardioverter-defibrillator.

If participants are unable to complete the resistance exercise set with continuous repetitions, an intra-set rest (“cluster” set) may be provided. This method produces comparable results to completing the set using traditional set structures (i.e., completing the prescribed repetition range in a set without rest) in healthy and clinical cohorts (133, 134).

In the setting where a participant is unable to complete a prescribed exercise, a suitable alternative that targets the same muscle group will be prescribed.

### Usual Care

Participants randomized into the usual care (control) group will continue with routine clinical care as directed by their treating medical team. They will also be instructed to continue with their usual daily activities and will not be restricted or asked to refrain from engaging in physical activity or exercise training. Participants allocated to the usual care group will be offered 4-months of telehealth exercise training (in adolescents and adults) or the physical activity intervention (in children) after their final 12-month testing session.

### Maintenance Phase

After 4-months of traditional exercise training, telehealth exercise training, or the physical activity program, participants in the exercise intervention groups will be encouraged to continue to engage in physical activity or exercise training at least two times a week. Adolescent and adult Fontan participants in the exercise intervention groups will be provided complimentary access to a local fitness facility to facilitate ongoing adherence. Children Fontan participants will be encouraged to join community sporting organizations and participate in a range of physical activities. The study team will contact participants every fortnight for the initial 2 months and every month after for the remaining duration of the study to provide remote support. Some people may receive up to 3 “booster” sessions delivered by an exercise professional to promote ongoing physical activity or exercise training participation.

### Education

To complement the exercise training and physical activity interventions, participants will also receive education on a variety of topics. This may include topics on understanding their congenital lesion, how to integrate physical activity into their daily routine, and nutrition and healthy eating. Education will be disseminated and delivered by information sheets and pre-recorded online videos.

### Safety and Adverse Events

Safety will be evaluated by reviewing the adverse events recorded in each group. All adverse events will be continuously recorded throughout the trial using a case report form. The severity of the reported adverse event (i.e., serious or non-serious) and the likelihood of the event being related to testing or the intervention will be evaluated.

### Adherence and Compliance

Adherence and compliance to exercise training will be monitored using various methods, including attendance to sessions, training logs, and HR monitors. In the F-FIT, adherence to the exercise training program will be considered as attending to 80% of the prescribed sessions—with attendance to at least 70% of sessions in the 4 weeks preceding the follow-up assessment visit. Non-adherent participants are defined as participants that attend <20% of the prescribed sessions, and partially adherent participants are considered as those who attend 20-79% of the prescribed sessions.

## Conclusion

Multiple factors influence aerobic exercise capacity; suboptimal preload appears to be the predominant factor impairing aerobic exercise capacity. Reduced ventricular filling is primarily associated with low lean mass, diastolic dysfunction, and abnormal pulmonary vascular development and function. Preliminary evidence shows exercise training is a safe and effective therapy for improving peak VO_2_ in people with a Fontan circulation. The F-FIT aims to provide high-quality evidence on the effects of physical activity and exercise training for increasing aerobic exercise capacity. A telehealth home-based exercise intervention will also be evaluated as a scalable and economical model of exercise training delivery. Furthermore, this multi-center randomized controlled trial will provide insight into the physiological changes associated with exercise training and unravel important pathophysiology.

## Author Contributions

DT drafted the exercise intolerance review and co-drafted the remaining sections of the manuscript. HG co-drafted the protocol sections of the manuscript. RC supervised the development of the protocol and manuscript. AM, DB, DC, DL, DT, JA, JC, NM, and RC contributed to the conception and design of the study. All authors critically reviewed the manuscript, contributed to study design, and approved the submission of the manuscript.

## Funding

This study was supported by a Grant from Additional Ventures, the National Heart Foundation of Australia Vanguard Grant (102277), and the Medical Research Future Fund—Cardiovascular Health Mission—Congenital Heart Disease Grant (ARGCHDG000016). DT was supported by the Paulette Isabel Jones Ph.D. Completion Scholarship.

## Conflict of Interest

The authors declare that the research was conducted in the absence of any commercial or financial relationships that could be construed as a potential conflict of interest.

## Publisher's Note

All claims expressed in this article are solely those of the authors and do not necessarily represent those of their affiliated organizations, or those of the publisher, the editors and the reviewers. Any product that may be evaluated in this article, or claim that may be made by its manufacturer, is not guaranteed or endorsed by the publisher.
